# A direct-drive GFP reporter for studies of tracheal development in *Drosophila*

**DOI:** 10.1080/19336934.2022.2030191

**Published:** 2022-01-30

**Authors:** Geanette Lam, Katherine Beebe, Carl S. Thummel

**Affiliations:** a Department of Human Genetics, University of Utah School of Medicine, Salt Lake City, UT, USA; b Molecular Medicine Program, University of Utah School of Medicine, Salt Lake City, UT, USA

**Keywords:** Tracheal development, trachea, GFP reporter, GAL4/UAS system

## Abstract

The *Drosophila* tracheal system consists of a widespread tubular network that provides respiratory functions for the animal. Its development, from ten pairs of placodes in the embryo to the final stereotypical branched structure in the adult, has been extensively studied by many labs as a model system for understanding tubular epithelial morphogenesis. Throughout these studies, a *breathless* (*btl*)*-GAL4* driver has provided an invaluable tool to either mark tracheal cells during development or to manipulate gene expression in this tissue. A distinct shortcoming of this approach, however, is that *btl-GAL4* cannot be used to specifically visualize tracheal cells in the presence of other GAL4 drivers or other UAS constructs, restricting its utility. Here we describe a direct-drive *btl-nGFP* reporter that can be used as a specific marker of tracheal cells throughout development in combination with any GAL4 driver and/or UAS construct. This reporter line should facilitate the use of *Drosophila* as a model system for studies of tracheal development and tubular morphogenesis.

## Introduction

The insect tracheal system consists of a complex branching network of tubes that extends throughout the body, providing the oxygen required for cellular function. Many studies have focused on tracheal development in *Drosophila* as a model for organogenesis and tubular morphogenesis, with implications for the development of more complex organs such as the mammalian lung and kidneys [1–4]. The tracheal system originates as ten pairs of placodes that invaginate into the embryonic body and connect to form a stereotypical branched structure. This structure refines during embryogenesis into two long dorsal tracheal trunks that run from the anterior to posterior ends of the developing larva. Regularly-spaced tubular branches extend laterally off these dorsal trunks and elaborate into a fine network of tracheal terminal cells that extend throughout the body. This tracheal system acts as the respiratory organ for the growing larva and is remodelled during metamorphosis to provide a similar function for the adult fly [1].

The predominant genetic tool used to characterize tracheal development and function is a GAL4 driver from the Hayashi lab that carries regulatory sequences from the *breathless* (*btl*) locus in *Drosophila* [5]. *Btl* encodes a Fibroblast Growth Factor (FGF) receptor that is expressed in the trachea and required for early tracheal development as well as the outgrowth of tracheal terminal cells in response to anoxia [6–8]. The *btl-GAL4* driver thus provides an ideal means of specifically targeting genetic rescue constructs or RNAi to tracheal cells. In addition, combining this driver with UAS-regulated reporter constructs provides the most commonly used method to specifically mark the tracheal system of the animal [5]. This approach has been widely used in many studies of tracheal development and morphology over the past 25 years, usually using *btl-GAL4* to drive a GFP reporter (*btl>GFP*) [e.g. 9–13]. A disadvantage of using this system, however, is that it is not possible to combine *btl>GFP* with *UAS-RNAi* transgenes that disrupt gene expression in other tissues since the RNAi construct will also be active in the tracheal system. A similar problem is encountered when the GAL4/UAS system is used to overexpress genes in different tissues. Accordingly, several labs have developed direct-drive fluorescent markers to follow tracheal development independently of the GAL4 system. These include *btl-GFP-moe* and *btl-RFP-moe* that carry fusions of fluorescent marker proteins to the actin binding domain of moesin [14,15]. This allows specific labelling of the actin cytoskeleton in developing tracheal cells and thus provides a useful marker of cellular morphology. In addition, a direct-drive *SRF-GFP* reporter has been constructed to specifically follow the morphology of tracheal terminal cells [16]. Here we describe a fusion of the *btl* promoter to a nuclear *GFP* reporter gene (*nGFP*). The restricted localization of nuclear GFP often results in a stronger fluorescent signal than cytoplasmic expression, thus facilitating cellular detection. Here we describe this *btl-nGFP* reporter and demonstrate that it provides a strong nuclear signal in developing tracheal cells. This construct should provide a useful tool for researchers interested in studying *Drosophila* tracheal development and function.

## Materials and methods

### *Construction of the* btl-nGFP *reporter transgene*

An *attB* attachment site for the PhiC31 integrase system was inserted into the pH-Stinger vector described by Barolo et al. [17]. This was accomplished by using the following primers to PCR amplify the *attB* sequence from a pUAST-attB template: ACGTCCTAGGGTCGACGATGTAGGTCACGG and GACTCCTAGGTCGACATGCCCGCCGTGACCG. This resulted in a ~ 300 bp fragment containing an *attB* site with AvrII restriction sites at the ends. This fragment was inserted into the single AvrII site in pH-Stinger to generate the pH-Stinger-attB vector [17].

Two primers were used to PCR amplify the second intron of the *btl* locus from the pCas4-*btl*-GAL4 plasmid (a generous gift from S. Hayashi) [5]: GAATTCTGCGACCTTGGCTTAAAGAC and CTCGAGTCAAATCGAGCGTTCTCCAG. This resulted in a 3.33 kb fragment with EcoRI and XhoI sites at the ends. This was inserted between the EcoRI and XhoI sites in the multiple cloning site of the pH-Stinger-attB vector to generate the *btl-nGFP* reporter construct. This was introduced into the *Drosophila* genome using the PhiC31 integrase system, targeting the construct into the *attP-3B* insertion site on the X chromosome (Bloomington stock number 9753; VK00038) [18]. Proper integration was confirmed by PCR amplification and DNA sequencing. The stock carrying this *btl-nGFP* reporter can be obtained from the Bloomington stock centre (stock # 93131): *y* [1] *w[1118] PBac[y[+mDint2] w[+mC] = btl-H-Stinger]VK00038*.

### Microscopy and imaging

Flies carrying the *btl-nGFP* reporter construct were transferred to bottles with standard grape juice agar caps supplemented with yeast paste. Embryos collected from these plates were harvested, washed with water, dechorionated for 1 minute using 50% bleach in PBS, washed again with water, and mounted in glycerol on slides for imaging with a Leica SP8 laser confocal microscope.

Individual living second and third instar larvae carrying the *btl-nGFP* construct were serially transferred to glycerol to remove food and then transferred to a slide containing a 3% agar base. The larvae were pressed with a cover slip and tape to minimize their movement during imaging, essentially as described [19]. Larvae were imaged using a Leica SP8 laser confocal microscope (Figures 2 and 3(a,c)) or a Zeiss Axioskop2 Plus microscope (Figure 3(b), Movie S1).

## Results & discussion

### *Construction of a transgenic* Drosophila *line that carries the* btl-nGFP *reporter*

**Figure 1.**
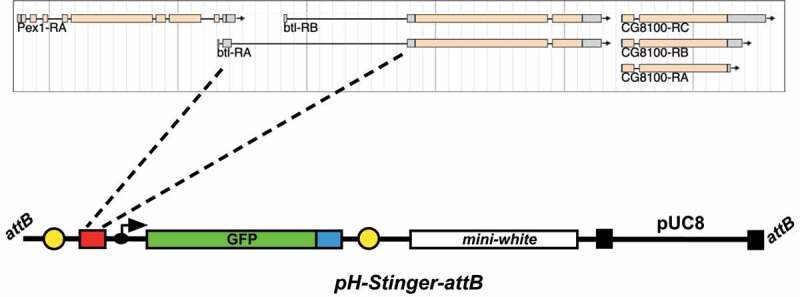
A direct-drive *btl-nGFP* reporter construct. A 3.3 kb region corresponding to the second intron of the *btlA* mRNA isoform was inserted into the pH-Stinger-attB vector for fly transformation. This region corresponds to the only large non-coding sequences within the *btl* locus. The pH-Stinger-attB construct is shown below in a linearized format with the *attB* sequences at the ends, representing its structure upon insertion into the *Drosophila* genome. The two gypsy transposon insulator elements (yellow), multiple cloning site (red), minimal promoter (black arrow), GFP reporter gene (green, GFP), nuclear localization signal (blue), *mini-white* marker gene (white), P element ends (black), and bacterial pUC8 plasmid are shown.

Our original goal was to fuse the same *btl* regulatory sequences contained in the *btl-GAL4* driver described by Shiga et al. [5] with a GFP reporter gene. In their description of this construct, the *btl* sequences were reported as an ‘approximately 3 kb EcoRI-NdeI fragment carrying the *btl* promoter’ [5]. The *Drosophila* reference genome sequence for 10 kb surrounding the *btl* locus, however, does not have a restriction fragment of this size. This is likely due to polymorphisms in the genomic DNA sequence of the stock used by Shiga et al. relative to the FlyBase reference genome. In addition, the *btl* gene is closely flanked on both sides by other protein-coding genes – the *Pex1* gene, which overlaps with the 5’ end of the *btl* gene, and *CG8100*, which lies ~350 bp downstream from the 3’ end of *btl* (Figure 1). We thus focused on the only large non-coding sequence within the *btl* locus, which corresponds to the second intron of the *btl-A* mRNA isoform (Figure 1). We used PCR to amplify sequences from the middle of the second exon of *btl-A* to the beginning of the third exon, encompassing the entire 3.3 kb region encoding the second intron of this transcript (Figure 1). Importantly, this fragment includes the ~400 bp region identified by Ohshiro and Saigo that contains important cis-regulatory elements that control *btl* expression in the tracheal system of *Drosophila* [20].

The pH-Stinger construct was described by Barolo et al. [17] as a tool to facilitate studies of gene regulation in *Drosophila*. It consists of a bacterial plasmid with *Drosophila* P-element ends for fly transformation and a *mini-white* reporter to identify transformants (Figure 1). In addition, the plasmid carries a multiple cloning site upstream from a minimal *Drosophila* promoter fused to a nuclear GFP reporter gene. Thus, an enhancer sequence inserted into the multiple cloning site will direct nGFP expression in a specific pattern that reflects its activity. The vector was further modified by inserting insulator elements from the *gypsy* transposon at either side of the GFP expression cassette. This provides a means of effectively suppressing the influence of neighbouring sequences on the expression of the GFP reporter. We modified the pH-Stinger vector by inserting a single copy of the *attB* targeting sequence for the PhiC31 integrase system [21]. This facilitates the use of the library of *attP*-carrying *Drosophila* strains for targeting plasmid constructs to specific regions of the fly genome [18]. Finally, we inserted the 3.3 kb fragment corresponding to the second intron of *btl* into the multiple cloning site of the pH-Stinger-attB plasmid to generate the *btl-nGFP* reporter. The pattern of nuclear GFP expressed by this construct should reflect the expression pattern dictated by the *btl* regulatory sequences. This final construct was integrated into the *Drosophila* genome on the X chromosome and used to characterize the pattern of nuclear GFP expression.

### *Characterization of the* btl-nGFP *expression pattern in* Drosophila

**Figure 2.**
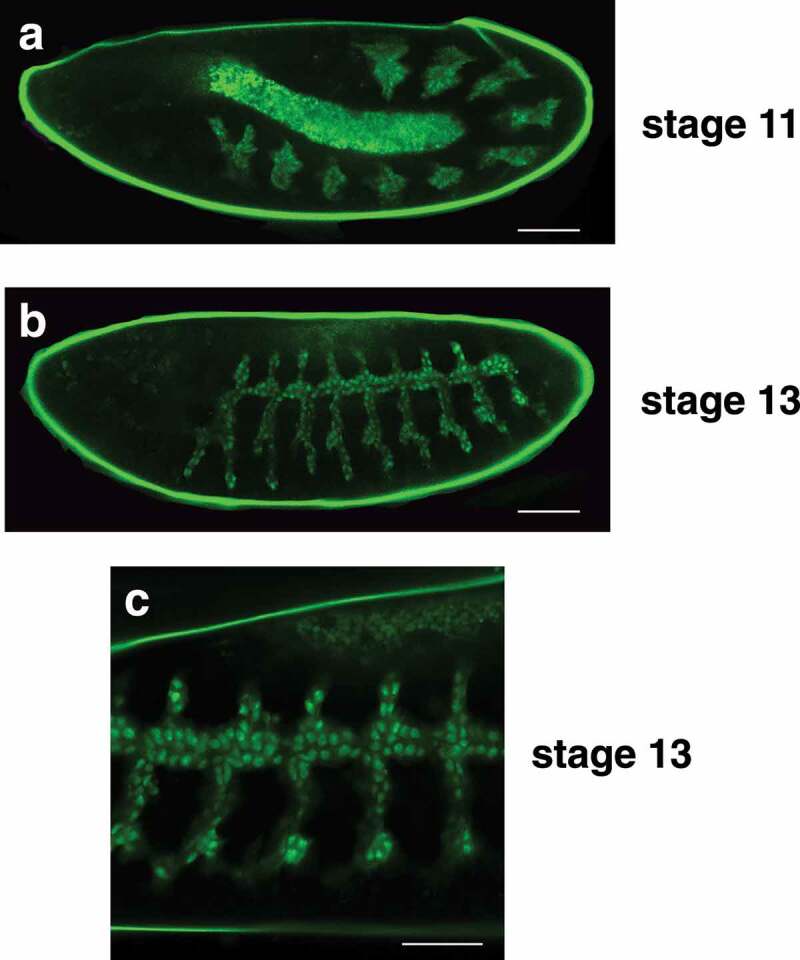
The *btl-nGFP* construct is expressed in the developing embryonic tracheal system. (a) A lateral view of a stage 11 embryo is depicted with the anterior end on the left. The *btl-nGFP* construct is expressed in the ten clusters of tracheal precursor cells that are distributed in a segmented pattern along the length of the germband extended embryo. The central amnioserosa is illuminated by background fluorescence. Scale bar = 50 µm. (b) A lateral view of a stage 13 embryo is depicted with the anterior end on the left. The *btl-nGFP* construct is expressed in the developing tracheal branches. Scale bar = 50 µm. (c) A higher magnification image is depicted of a stage 13 embryo viewed from the lateral perspective with the anterior end on the left. The nuclear localization of the *btl-nGFP* reporter can be clearly seen in the developing tracheal system and newly-fused dorsal trunk branches. Scale bar = 30 µm.

The tracheal system develops from pairs of ectodermal placodes arranged in a segmented pattern along the lateral sides of the stage 10 embryo. These cells invaginate to form ten tracheal pits that expand internally to form the precursors of the branched tracheal system. During stage 13, the adjacent dorsal trunk branches in each segment fuse to form a continuous network of tracheal precursor cells that display a characteristic branching pattern along the sides of the embryo. This epithelial network continues to undergo tubular morphogenesis during the subsequent stages of embryogenesis, eventually forming the elaborate tracheal system of the first instar larva. As expected, the *btl-nGFP* reporter is expressed throughout embryonic tracheal development, revealing the ten clusters of tracheal precursor cells in the stage 11 germband extended embryo (Figure 2(a)). Dorsal trunk fusion and tracheal branch outgrowth is also evident in stage 13 embryos (Figure 2(b)). A higher magnification image reveals that the majority of the GFP from the *btl-nGFP* reporter resides in the nucleus, as expected (Figure 2(c)).

**Figure 3.**
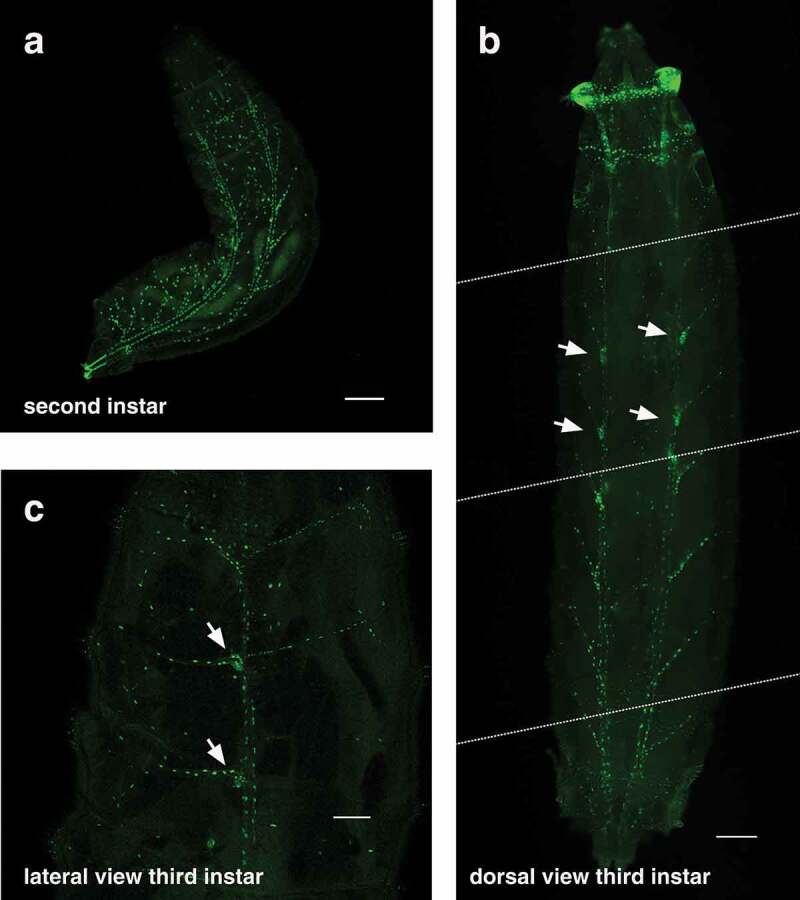
The *btl-nGFP* reporter is expressed throughout the tracheal system of second and third instar larvae. (a) A dorsal view of a second instar larva is depicted with the anterior end at the top and the posterior spiracles marked with GFP in the lower left. The *btl-nGFP* reporter can be seen throughout the branched tracheal system. Some background fluorescence is seen in the larval fat body. Scale bar = 100 µm. (b) A dorsal view of a third instar larva is depicted with the anterior end at the top. This panel was constructing by aligning four images captured on the microscope from a single animal. The dashed white lines represent the boundaries of each image. *btl-nGFP* expression can be seen throughout the branched tracheal system. The spiracular branch tracheoblasts, which are known to express *btl*, are marked by arrows. Scale bar = 100 µm. (c) A lateral view of a third instar larva is depicted with the anterior end at the top. *btl-nGFP* expression can be seen throughout the branched tracheal system. The spiracular branch tracheoblasts are marked by arrows. Scale bar = 300 µm.

The elaborate structure of the tracheal system is revealed in the second instar larva by *btl-nGFP* expression (Figure 3(a)). The pair of tracheal trunks can be seen along the dorsal surface of the animal with regularly-spaced tracheal branches that extend laterally down the sides. It is more difficult to visualize the tracheal system in third instar larvae because of their large size, but the *btl-nGFP* reporter is expressed in nuclei throughout the tracheal branches of these animals (Figure 3(b,c), Movie S1). The spiracular branch tracheoblasts can also be seen in these images (Figure 3(b,c), arrows). These small clusters of imaginal cells are located adjacent to the dorsal trunk in third instar larvae and will develop into the adult tracheal tubes and spiracles during metamorphosis [22,23]. Finally, the *btl-nGFP* reporter can be used to visualize the tracheal system in living animals, providing a dynamic approach for studies of tracheal development and function (Movie S1).

In conclusion, we have generated a direct-drive *btl-nGFP* reporter that directs high levels of nuclear GFP expression throughout the tracheal system of *Drosophila*. This construct provides a valuable tool for researchers studying tubular morphogenesis as well as tracheal development and function. The *btl-nGFP* reporter is available from the Bloomington stock centre (stock # 93131)

## Supplementary Material

Supplemental Material

## References

[1] Hayashi S, Kondo T. Development and function of the Drosophila tracheal system. Genetics. 2018;209(2):367–380. 29844090 10.1534/genetics.117.300167 PMC5972413

[2] Kotini MP, Mae MA, Belting HG, et al. Sprouting and anastomosis in the Drosophila trachea and the vertebrate vasculature: similarities and differences in cell behaviour. Vascul Pharmacol. 2019;112:8–16. 30423447 10.1016/j.vph.2018.11.002

[3] Schottenfeld J, Song Y, Ghabrial AS. Tube continued: morphogenesis of the Drosophila tracheal system. Curr Opin Cell Biol. 2010;22(5):633–639. 20739171 10.1016/j.ceb.2010.07.016 PMC2948593

[4] Metzger RJ, Krasnow MA. Genetic control of branching morphogenesis. Science. 1999;284(5420):1635–1639. 10383344 10.1126/science.284.5420.1635

[5] Shiga Y, Tanaka-Matakatsu M, Hayashi S. A nuclear GFP/ß-galactosidase fusion protein as a marker for morphogenesis in living Drosophila. Dev Growth Differ. 1996;38:99–106.

[6] Glazer L, Shilo BZ. The Drosophila FGF-R homolog is expressed in the embryonic tracheal system and appears to be required for directed tracheal cell extension. Genes Dev. 1991;5(4):697–705. 1849109 10.1101/gad.5.4.697

[7] Klambt C, Glazer L, Shilo BZ. breathless, a Drosophila FGF receptor homolog, is essential for migration of tracheal and specific midline glial cells. Genes Dev. 1992;6(9):1668–1678. 1325393 10.1101/gad.6.9.1668

[8] Ghabrial A, Luschnig S, Metzstein MM, et al. Branching morphogenesis of the Drosophila tracheal system. Annu Rev Cell Dev Biol. 2003;19:623–647. 14570584 10.1146/annurev.cellbio.19.031403.160043

[9] Jazwinska A, Ribeiro C, Affolter M. Epithelial tube morphogenesis during Drosophila tracheal development requires Piopio, a luminal ZP protein. Nat Cell Biol. 2003;5(10):895–901. 12973360 10.1038/ncb1049

[10] Jiang L, Crews ST. Dysfusion transcriptional control of Drosophila tracheal migration, adhesion, and fusion. Mol Cell Biol. 2006;26(17):6547–6556. 16914738 10.1128/MCB.00284-06 PMC1592841

[11] Metzstein MM, Krasnow MA. Functions of the nonsense-mediated mRNA decay pathway in Drosophila development. PLoS Genet. 2006;2(12):e180. 17196039 10.1371/journal.pgen.0020180 PMC1756896

[12] Roy S, Hsiung F, Kornberg TB. Specificity of Drosophila cytonemes for distinct signaling pathways. Science. 2011;332(6027):354–358. 21493861 10.1126/science.1198949 PMC3109072

[13] Massarwa R, Schejter ED, Shilo BZ. Apical secretion in epithelial tubes of the Drosophila embryo is directed by the Formin-family protein Diaphanous. Dev Cell. 2009;16(6):877–888. 19531358 10.1016/j.devcel.2009.04.010

[14] Kato K, Chihara T, Hayashi S. Hedgehog and Decapentaplegic instruct polarized growth of cell extensions in the Drosophila trachea. Development. 2004;131(21):5253–5261. 15456724 10.1242/dev.01404

[15] Ribeiro C, Neumann M, Affolter M. Genetic control of cell intercalation during tracheal morphogenesis in Drosophila. Curr Biol. 2004;14(24):2197–2207. 15620646 10.1016/j.cub.2004.11.056

[16] Nikolova LS, Metzstein MM. Intracellular lumen formation in Drosophila proceeds via a novel subcellular compartment. Development. 2015;142(22):3964–3973. 26428009 10.1242/dev.127902 PMC6517834

[17] Barolo S, Carver LA, Posakony JW. GFP and beta-galactosidase transformation vectors for promoter/enhancer analysis in Drosophila. Biotechniques. 2000;29(4):727–732. 10.2144/00294bm10 11056799

[18] Venken KJ, Bellen HJ. Genome-wide manipulations of Drosophila melanogaster with transposons, Flp recombinase, and PhiC31 integrase. Methods Mol Biol. 2012;859:203–228. 22367874 10.1007/978-1-61779-603-6_12

[19] Weiner AT, Seebold DY, Torres-Gutierrez P, et al. Endosomal Wnt signaling proteins control microtubule nucleation in dendrites. PLoS Biol. 2020;18(3):e3000647. 32163403 10.1371/journal.pbio.3000647 PMC7067398

[20] Ohshiro T, Saigo K. Transcriptional regulation of breathless FGF receptor gene by binding of TRACHEALESS/dARNT heterodimers to three central midline elements in Drosophila developing trachea. Development. 1997;124(20):3975–3986. 9374395 10.1242/dev.124.20.3975

[21] Fish MP, Groth AC, Calos MP, et al. Creating transgenic Drosophila by microinjecting the site-specific phiC31 integrase mRNA and a transgene-containing dono*r plasmid*. Nat Protoc. 2007;2(10):2325–2331. 17947973 10.1038/nprot.2007.328

[22] Pitsouli C, Perrimon N. Embryonic multipotent progenitors remodel the Drosophila airways during metamorphosis. Development. 2010;137(21):3615–3624. 20940225 10.1242/dev.056408 PMC2964094

[23] Weaver M, Krasnow MA. Dual origin of tissue-specific progenitor cells in Drosophila tracheal remodeling. Science. 2008;321(5895):1496–1499. 18669822 10.1126/science.1158712 PMC3999966

